# Leukocyte super-resolution via geometry prior and structural consistency

**DOI:** 10.1117/1.JBO.25.10.106501

**Published:** 2020-10-05

**Authors:** Xia Hua, Yue Cai, You Zhou, Feng Yan, Xun Cao

**Affiliations:** Nanjing University, School of Electronic Science and Engineering, Nanjing, China

**Keywords:** imaging, microscopy, deep learning, super resolution

## Abstract

**Significance**: Researchers have made great progress in single-image super-resolution (SISR) using deep convolutional neural networks. However, in the field of leukocyte imaging, the performance of existing SISR methods is still limited as it fails to thoroughly explore the geometry and structural consistency of leukocytes. The inaccurate super-resolution (SR) results will hinder the pathological study of leukocytes, since the structure and cell lineage determine the types of leukocyte and will significantly affect the subsequent inspection.

**Aim**: We propose a deep network that takes full use of the geometry prior and structural consistency of the leukocyte images. We establish and annotate a leukocyte dataset, which contains five main types of leukocytes (basophil, eosinophil, monocyte, lymphocyte, and neutrophil), for learning the structure and geometry information.

**Approach**: Our model is composed of two modules: prior network and SR network. The prior network estimates the parsing map of the low-resolution (LR) image, and then the SR network takes both the estimated parsing map and LR image as input to predict the final high-resolution image.

**Result**: Experiments show that the geometry prior and structural consistency in use obviously improves the SR performance of leukocyte images, enhancing the peak-signal-to-noise ratio (PSNR) by about 0.4 dB in our benchmark.

**Conclusion**: As proved by our leukocyte SR benchmark, the proposed method significantly outperforms state-of-the-art SR methods. Our method not only improves the PSNR and structural similarity indices, but also accurately preserves the structural details of leukocytes. The proposed method is believed to have potential use in the wide-field cell prescreening by simply using a low-power objective.

## 1 Introduction

Image super-resolution (SR), which is a classic low-level task in the field of computer vision, aims at recovering high-resolution (HR) image from a given low-resolution (LR) image. Obviously, image SR is an ill-posed inverse problem since an LR image may correspond to many HR candidates. Recently, convolutional neural network (CNN) has been introduced into the image SR problem. This powerful technology has brought new life to SR algorithms.^1^^–^^8^

### 1.1 Image Super-Resolution

Image SR has become an important branch of computer vision tasks. It can be categorized into four types according to Yang’s work:^9^ prediction models, edge-based methods, image statistical methods, and patch-based (or example-based) methods. Among them, patch-based methods, especially those utilizing deep CNN models, achieve better performance than the other three methods. The well-known algorithm called bicubic interpolation belongs to the first, which is able to calculate SR images in a very short time. However, bicubic interpolation loses most high-frequency information in LR images.

Dong et al.’s work^1^ first introduced a deep CNN to the SR algorithm, in which a three-layer CNN (called SRCNN) learns the mapping between LR and HR patches based on large external datasets such as 91 images and Imagenet.^10^ Later, Kim et al.^2^ proposed a very deep convolution network (called VDSR) to capture deeper features of the input image. They also introduced a skip-connection between the input image and the final feature layer, which enables a higher learning rate and mitigates gratitude explosion/vanishing.^11^ There are also some studies focusing on making full use of geometry prior such as Chen et al.’s work.^8^ Their proposed model first generates a coarse SR image using CNN, then the image features and the landmark parsing maps are estimated simultaneously using two separate networks. Yang et al.^12^ reviewed representative deep learning-based single-image super-resolution (SISR) methods and group them into two categories according to their contributions to two essential aspects of SISR: the exploration of efficient neural network architectures for SISR and the development of effective optimization objectives for deep SISR learning. Finally, both the features and the maps are concatenated in the channel dimension and sent to a fine SR network to recover the HR image, which shows remarkable improvement over the other none-prior methods in the field of human face SR.

### 1.2 Deep Learning in Pathology

Since CNN-based methods have achieved remarkable success in the field of computer vision, it is not surprising that deep learning is about to be used in pathology. Researchers have witnessed the obvious trend of integration of deep learning and pathology, particularly in the field of cell segmentation, cell classification, tissue staining, cancer diagnosis, etc. Koyuncu et al.^13^ applied a multitask deep regression model for cell detection in images acquired with inverted microscopy. Fu et al.^14^ designed an 11-layer CNN for segmentation of histological images, particularly those with Masson’s trichrome stain. Microscopy SR has also been developed under deep learning frameworks. Rivenson et al.^15^ proposed a deep neural network to super-resolve Masson’s trichrome stained lung tissue, Masson’s trichrome stained kidney tissue, H&E stained breast tissue, and so on. They also proposed a mobile-phone-version SR model,^16^ which can correct distortion introduced by mobile-phone-based microscopes and generate high-resolution tissue sample images. de Haan et al.^17^ reviewed some of these emerging applications of deep learning ranging from image transformations between microscopic imaging systems to adding new capabilities to existing imaging techniques, as well as solving various inverse problems based on microscopy image data. Nevertheless, these aforementioned methods are more concerned tissue-level SR than cell-level SR.

### 1.3 Leukocyte Super-Resolution

Recovering high-frequency context in leukocytes is much more challenging since a leukocyte is much smaller than tissues and consists of many different types with tiny differences, which means SR reconstruction should be done precisely and carefully without changing the original shape. Here, we propose a prior-embedded SR network specific for leukocytes. Adhesion of the nucleus in leukocytes is an important criterion for leukocyte recognition. In order to preserve the adhesion information during super-resolving, we introduce the geometry prior, which constrains the structural consistency of the nucleus, cytoplasm, and backgrounds in leukocyte images. Our model can be divided into two parts: prior network and SR network. The prior network generates a three-channel parsing map by predicting the geometry information of a given LR leukocyte image. The SR network takes both the LR image and parsing map as input and finally generates a visually pleasing HR image. Experiments have demonstrated that our method outperforms none-prior SR methods mathematically and visually. It should be pointed out that our model can be easily transformed into other kinds of pathology SR tasks, including, e.g., holography, dark-field, and fluorescence.

## 2 Method

Given an input LR image of size W×H, we first upsample it to the size of the final HR image sW×sH using bicubic interpolation, where s means the scale factor. With this simple trick, the network earns a relatively good starting point, making the training process converge faster. It also keeps pixel-to-pixel correspondence between input and output. In the following sections, we mark $I^{\mathrm{HR}}$ and $I^{\mathrm{LR}}$ as HR and LR image, respectively, with both having size sW×sH.

### 2.1 Prior Network

In the proposed approach, we use parsing maps as the cell’s geometry prior. So prior estimation equals semantic segmentation. The network takes $I^{\mathrm{LR}}$ as input and estimates a three-channel parsing map $I^{M}$, which represents the region of the nucleus, cytoplasm, and backgrounds, respectively.

We follow the design philosophy of U-net structure as illustrated in Ref. 18, which has been successfully utilized in the field of microscopy image segmentation. Nevertheless, there are still some differences between U-net and our proposed network. First, in the U-net, spatial size of the feature maps keeps shrinking in the encoder module and expanding in the decoder module, and we use zero-padding to keep spatial size unchanged during convolutions in the proposed network. Second, we half the channels of feature maps in U-net, since we found this light version still obtained good results. There also exist some semantic segmentation models more powerful than U-net such as Refs. 19–23. These methods focus on large natural image datasets such as VOC2012^24^ and COCO,^25^ which contain tens of semantic class and dense detection targets. We use the relatively small U-net for the consideration of computation speed.

### 2.2 SR Network

**Fig. 1:**
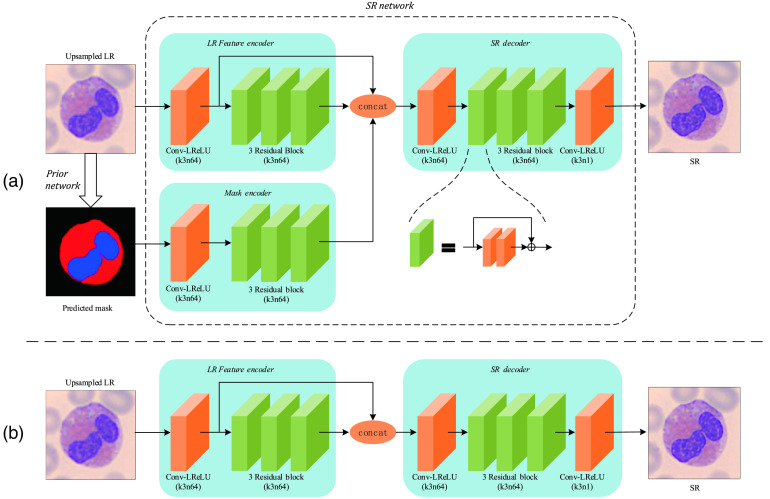
Network structure of our proposed model. The orange block indicates a convolutional layer followed by a Leaky ReLU. The green block indicates a residual block containing two continuous orange blocks and a skip connection. “k3n64” indicates the kernel size is 3×3, the feature map number is 64. The prior network is abridged since the structure almost equals U-net. (a) The full structure of our proposed model. (b) The structure of our proposed model without prior branch.

The architecture of our SR network is illustrated in Fig. 1(a). Our network can be divided into the following parts: LR feature encoder, mask encoder, and SR decoder.

The basic unit of our SR network is residual block similar to enhanced deep residual networks for single image super-resolution (EDSR).^6^ The block is built by a sequence of two convolution layers, and each convolution layer is followed by a Leaky rectified linear unit (ReLU). Batch normalization (BN) is discarded because it may break the diversity of images.

Inspired by SRGAN,^5^ we employ feature extractor on the input image. It contains a single conv layer followed by Leaky ReLU. Thus, the input $I^{\mathrm{LR}}$ with 1 channel will be transformed into a 64-channel shallow feature map. The LR feature encoder contains three stacked residual blocks, which further encodes shallow features into deep features. $I^{M}$ generated by a prior network is sent to a mask encoder network. Note that mask encoder has the same depth as the LR feature encoder. We design such a structure for the balance of both features’ depths. Then shallow features and deep features of the image, along with features of the mask, are stacked together and sent to the SR decoder, generating the final $I^{\mathrm{SR}}$.

All the convolutional layers generate features with the same channels except for the last one, and all the nonlinear layers are set as Leaky ReLU with a negative slope of 0.2. Note that a more stacked residual block may extract deeper features and result in better performance. Here, we use a relatively shallow residual block for computation speed.

### 2.3 Loss Functions

As described above, we have two mapping functions to be optimized. One is for generating the mask $I^{M}$ from the LR image $I^{\mathrm{LR}}$; $\theta_{1}$, another is for generating the SR image $I^{\mathrm{SR}}$ given the LR image $I^{\mathrm{LR}}$ and the corresponding mask $I^{M}$

$$I^{M}=f_{1}(I^{\mathrm{LR}};\theta_{1}),$$ (1)

$$I^{\mathrm{SR}}=f_{2}(I^{\mathrm{LR}},I^{M};\theta_{2})=f_{2}[I^{\mathrm{LR}},f_{1}(I^{\mathrm{LR}};\theta_{1});\theta_{2}].$$ (2)

Obviously, our model contains two corresponding losses: one for SR and another for semantic segmentation. Let ${\hat{I}}^{\mathrm{SR}}$ and ${\hat{I}}^{M}$ be the ground truth, and the overall loss function can be written as

L=1N∑i=1N[LSR(I^iSR−IiSR)+λLprior(I^iM−IiM)], (3)

where N is the number of training samples in each batch and λ is the coefficient balancing the two losses. Here, we set λ=1. For $L_{\text{prior}}$, we follow the binary cross entropy loss used in U-net.^18^ Since training with the optimization of the mean square error often results in over-smooth details, we use the more-robust loss function proposed in LapSRN^7^

$$L_{\mathrm{SR}}(\cdot )=\rho (\cdot ),$$ (4)

where $\rho (x)=\sqrt{x^{2}+\epsilon^{2}}$ is the Charbonnier penalty function.^26^ We set $\epsilon =10^{-3}$ empirically.

In summary, the total loss function can be written as

L=1N∑i=1N[ρ(I^iSR−IiSR)+BCE(I^iM−IiM)]. (5)

### 2.4 Implementation Details

In order to maintain the structural consistency, we do not crop them into small patches but take the full image as input. All images are converted to the YCbCr format. Our network only super-resolves the luminance channel, whereas the other two channels are upscaled by bicubic interpolation.

We set 3×3 as the size of all convolutional layers except the final one, whose kernel size is 1×1. For all convolutional layers, we adopt zero-padding to fix the image size. In the SR network, all convolutional layers produce feature maps with 64 filters, while the last one produces one-channel images. It is worth noting that the output of the initial feature extractor, the LR feature encoder, and mask encoder are concatenated in the channel dimension and sent into the SR decoder, so the input of the SR decoder network has 192 filters.

### 2.5 Training

We set the size of a minibatch as 8 considering computation complexity and convergence speed. We implement our network with the Pytorch-1.4 and update the parameters with the Adam optimizer.^27^ The learning rate is set to $10^{-4}$. We iterate parameters for 100 epochs on two NVIDIA GTX 1080 graphics processing units (GPUs), which take roughly 6 h for training.

The pretraining strategy eases the difficulty of training large, multitask models. It has also been used in some SR works or video SR works.^5^^,^^28^^–^^30^ In our early experiments, the mask predicted by a model without pretraining is precise enough and no much worse than that using pretraining. Thus, we choose end-to-end training, which learns the parameters of the prior network and SR network simultaneously.

## 3 Experiments

### 3.1 Dataset

We capture 863 leukocyte images as our dataset in which 759 were set as the training set, 52 were set as the validation set, and the other 52 were set as the testing set. The dataset contains five basic types of leukocytes: basophil, eosinophil, monocyte, lymphocyte, and neutrophil. Each image contains a single leukocyte observed with 100×/1.25  N.A. oil-immersion objective lens. The original images (with sizes of 224×224  pixels) are set as HR images, and the LR images were obtained by down-sampling HR images with MATLAB’s function imresize.

**Fig. 2:**
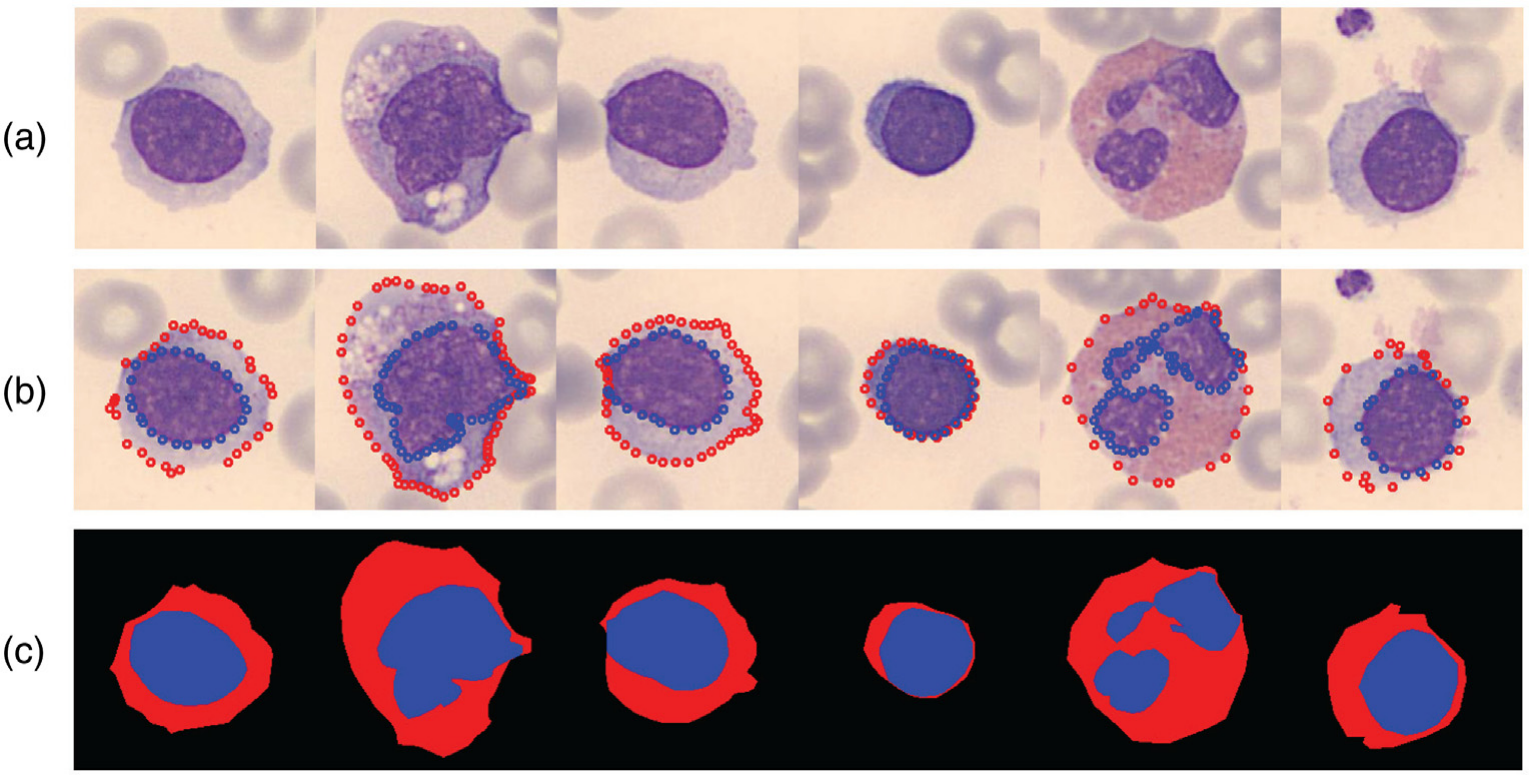
Some example images in our leukocyte dataset: (a) original leukocyte images and (b) ground-truth landmarks marked with LabelMe. The red circle represents the region of cytoplasm, and the blue circle represents the region of the nucleus. (c) Generated parsing maps according to the landmarks of (b).

We mark the parsing map with LabelMe tools.^31^ First, we handcraft several landmarks on the boundaries of the cytoplasm and nucleus. Then we connect these landmarks in order to create a closed polygon, which is also known as the parsing maps. We mark the nucleus as blue, cytoplasm as red, and background as black. Some examples are shown in Fig. 2.

### 3.2 Effects of Priors

Since our network can be divided into two parts, we easily obtain a none-prior SR network by removing the prior network and mask encoder, just as in Fig. 1(b). We mark the none-prior network as none-prior and the prior-contained network as geometry-prior. We train both the networks on our leukocyte dataset and test the peak-signal-to-noise ratio (PSNR) on the luminance channel. Table 1 shows the result of the above experiment. It can be seen that adding prior to the model significantly improves the performance of SR for leukocytes. It directly proves the power of using priors. The parsing map is available to guide the network to recover corresponding details in a specific region.

**Table 1:** Comparisons between none-prior and geometry-prior models on our dataset. Here, we only show the PSNR results of the experiments.

	None-prior	Geometry-prior
Leukocyte	39.5252	40.0031

### 3.3 Results of Segmentation

**Fig. 3:**
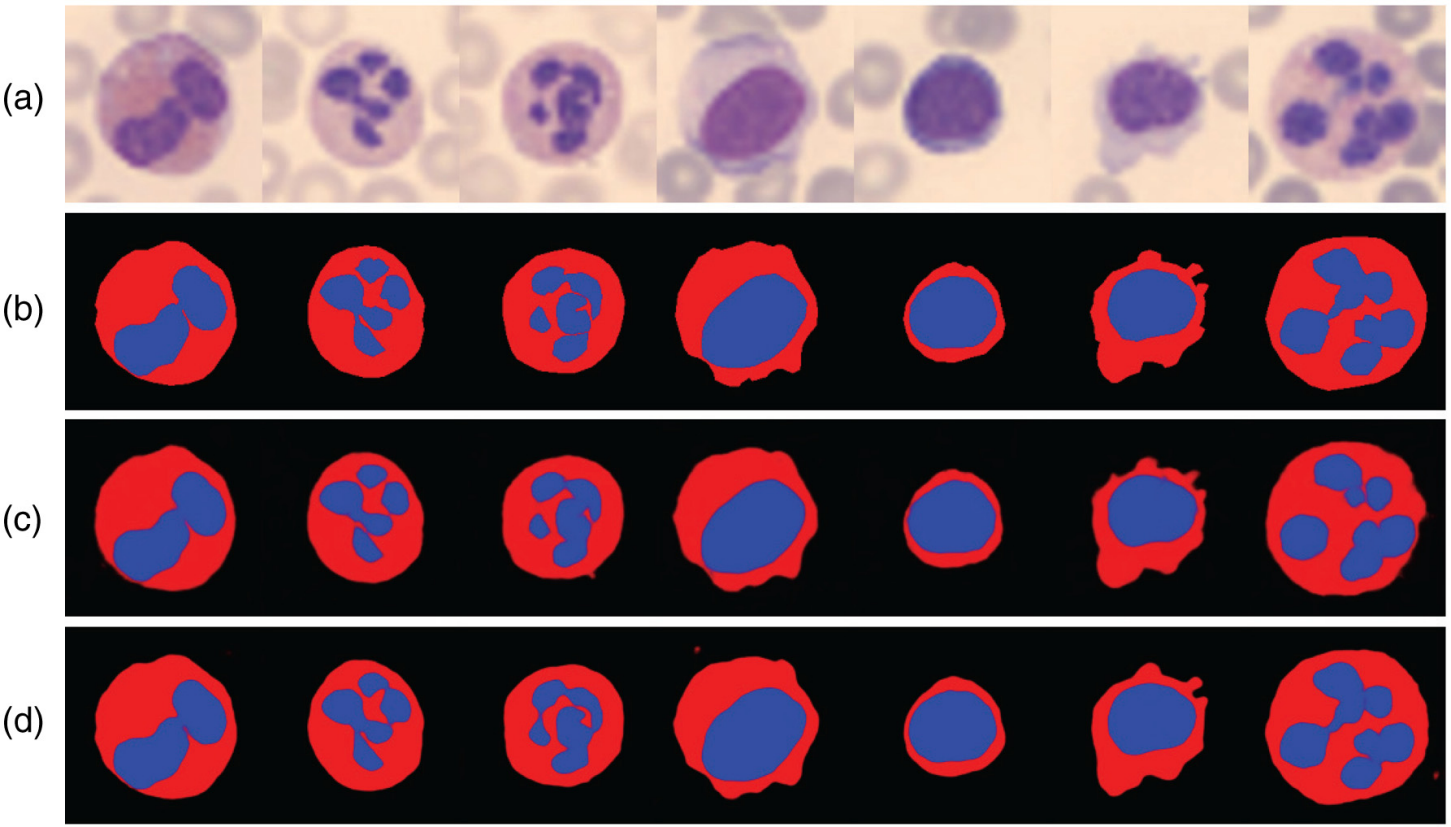
Results of parsing map prediction: (a) LR images. We only show the images down-sampled with scale 8×. (b) Ground-truth parsing maps, (c) predicted parsing maps at a scale of 8×, and (d) predicted parsing maps at a scale of 4×.

The results of parsing map estimation are shown in Fig. 3, and the ground truth parsing maps are marked by anchors. As shown, the predicted parsing maps are almost as precise as the ground truth, especially with a down-sampling scale 4×. Notice that parsing maps predicted from images down-sampled with a scale 8× are basically similar to that with a scale 4×. This indicates that our prior network is able to predict parsing maps in very LR images.

We do not compare our prior network with other segmentation models for two reasons: (1) we build a prior network not for reaching the state-of-the-art segmentation results, but for assisting the SR task. (2) The ground truth parsing maps of our dataset are not annotated pixel-by-pixel and are not suitable for any pixel-level metrics.

### 3.4 Comparing with the other Methods

We compare our method with other SR methods mentioned above. Since our network works only when prior masks are available, we make comparison on our 52 leukocyte images described in Sec. 3.1. We compute the PSNR^32^ and structural similarity (SSIM)^33^ between super-resolved images and ground-truth in a float32 format. For SRCNN, we use the most powerful version 9-5-5. For SRResNet, we use SRResNet-16 version (containing 16 residual blocks). EDSR^6^ is excluded here, because it uses far more filters and residual blocks than the other methods. For fair comparison, we reimplement them on Pytorch-0.3rc and ensure they have a similar performance on natural images. We conduct experiments on two down-sampling scales, 4× and 8×.

In order to maintain the consistency of the experimental results, several networks participating in the comparison are trained with the same parameter settings as our network. The learning rate is set to $10^{-4}$ and then decayed by a factor of 2 every 10 epochs. The size of the minibatch is set to 8 and the Adam optimizer is chosen as the optimizer with $\beta_{1}=0.9$. The number of epochs is set to 100, except for SRGAN and SFTGAN,^34^ because training of a generative adversarial network (GAN)-based network is more difficult to converge. We set the number of epochs for SFTGAN to 200. Feature maps used for SRGAN and SFTGAN are obtained with a pretrained 19-layer VGG network provided by torchvision.models.vgg19.

**Fig. 4:**
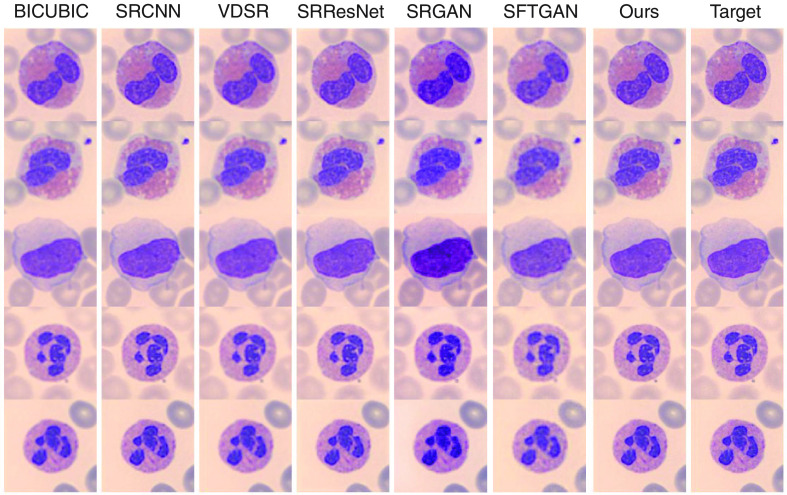
Qualitative comparisons (scale 4×). Readers are advised to zoom in for a better view (save image, then zoom in using a photo reader program).

**Fig. 5:**
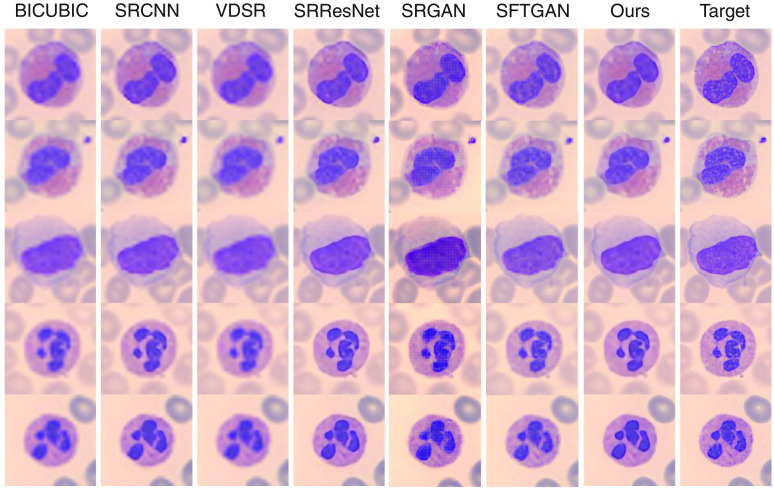
Qualitative comparisons (scale 8×). Readers are advised to zoom in for a better view.

Qualitative comparisons are shown in Table 2. Some super-resolved results are also shown in Figs. 4 and 5. To our great surprise, at scales both 4× and 8×, VDSR achieves very poor performances whose PSNR is even lower than SRCNN. The reason may be VDSR’s long-plain structure. The over-long convolutional sequence blocks effective gradient flowing, resulting in gradient exploding/vanishing. As we can see, the color and texture information in the microscopic image is relatively small, the influence of BN artifacts in the result of SRGAN is relatively high. SFTGAN improved the high-frequency details and handles the BN artifacts quite well. However, GAN-based methods tend to generate an unrealistic texture, which is not a good thing for medical images because morphological examination of peripheral blood cells relies heavily on image texture and structure information.

**Table 2:** Benchmark results with bicubic down-sampling models. PSNR and SSIM values are both calculated for scaling factors of 4× and 8×. Bold/italics values indicate the best/second best performance.

		Bicubic	SRCNN	VDSR	SRResNet	SRGAN	SFTGAN	Ours (NP)	Ours (GP)
4×	PSNR	36.6830	37.8846	36.6831	38.4418	36.1347	35.9718	**39.5252**	*39.9251*
	SSIM	0.9714	0.9775	0.9714	0.9785	0.9421	0.9238	**0.9832**	*0.9834*
8×	PSNR	31.1077	31.4251	31.1073	33.7853	31.4918	31.3653	**34.4041**	*34.9352*
	SSIM	0.9239	0.9314	0.9241	0.9498	0.9156	0.8566	**0.9565**	*0.9572*

Our network significantly surpasses all the other SR methods, either on PSNR or on SSIM. First, the none-prior version has significantly surpassed the state-of-the-art method SRResNet by about 1 dB at scale 4× and 0.6 dB at scale 8×, which means a very large improvement. It should be noticed that SRResNet has 16 residual blocks, which is much more than ours (6 blocks in total in the SR branch). We attribute this improvement to the suitable design of our model’s structure. Our final geometry-prior model further surpasses the none-prior version by about 0.4 dB at scale 4× and 0.5 dB at scale 8×, which has been shown in the previous experiments. The result has shown that our proposed model has superiority both in structure and in prior utilization.

**Fig. 6:**
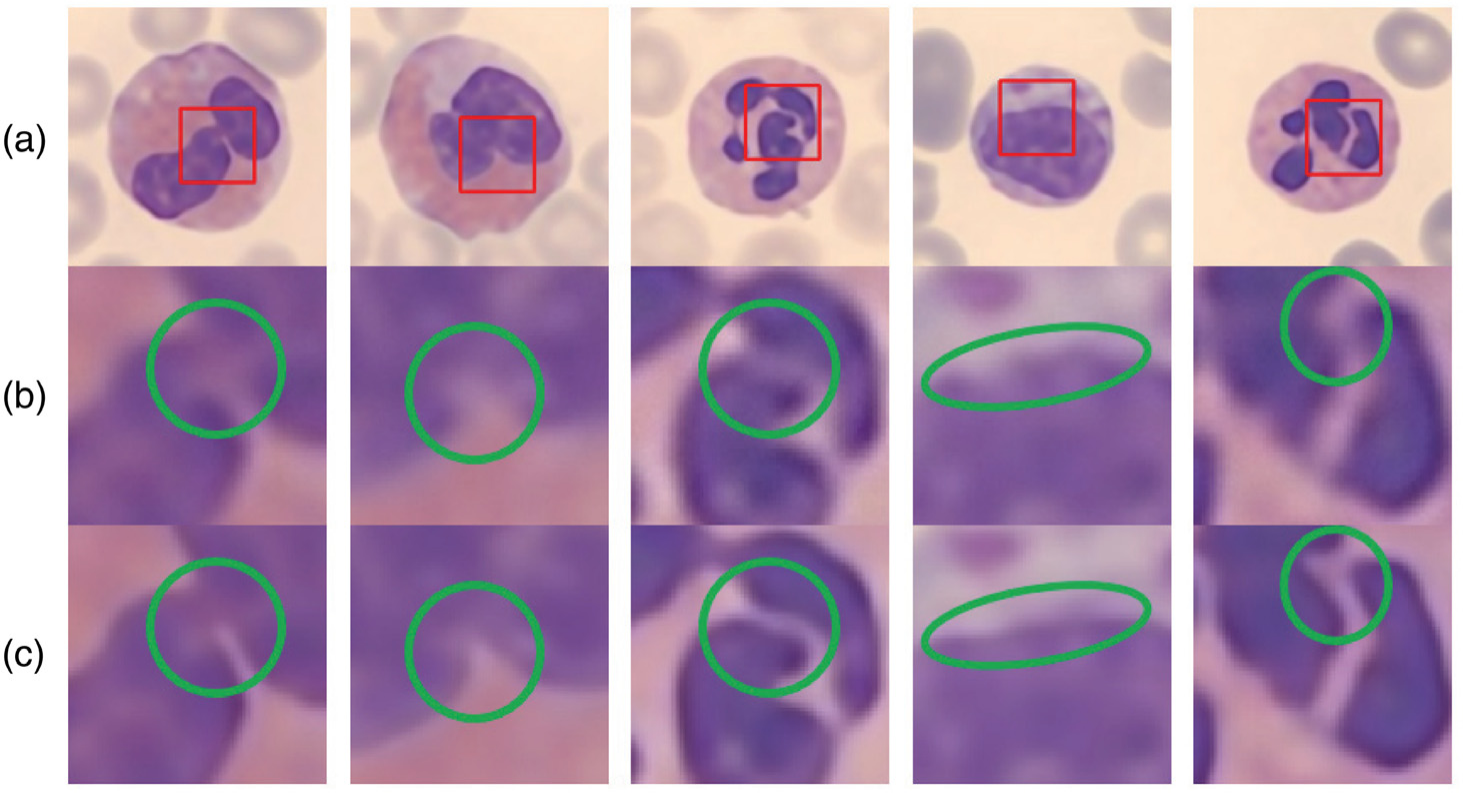
Qualitative comparisons at detail (scale 8×). Readers are advised to zoom in for a better view. (a) The full-size image generated by our method. The red bounding box indicates the region to be compared below. (b) Region of red bounding box in image generated by SRResNet. (c) Region of red bounding box in image generated by our method. The green circle indicates the region with significantly different contexts between the two methods.

An image super-resolved by SRResNet looks similar to that by our method at scale 8×. But if we take a closer look (Fig. 6), our method preserves shaper edges in many instances, such as cytoplasm’s boundary, joints between two nuclei, and a gully in the nucleus. This result shows that our model can recover more refined details than SRResNet in extreme situations. The comparison at scale 8× has practical significance since it is quite difficult for hospitals or research institutes in resource-limited areas to get high standard microscopes. Our method can mitigate this difficulty computationally, improving the quality of leukocyte images more significantly than other methods.

We have not tested our proposed method on the natural image benchmark since natural images contain much more semantic classes and more complex regions. As mentioned above, readers are welcomed to test our method on natural image datasets by replacing U-net with a more powerful network if interested.

## 4 Conclusion

In this paper, we proposed a deep end-to-end trainable SR network for leukocytes. The key component of the proposed method is the prior network, which estimates the parsing map of the leukocyte. The estimated parsing map and LR images were both sent to the SR network, and finally HR images are reconstructed. Experimental results have shown that the prior network improved the SR performance, enhancing the PSNR by about 0.4 dB compared with the none-prior network in our benchmark. In addition, our model achieved superiority over state-of-the-art methods both quantitatively and qualitatively. We have proposed and demonstrated that a prior-embedded deep neural network can improve optical microscopy imaging of leukocytes. For LR images synthesized from high-resolution images, this mapping process can be considered a simple averaging. For the mapping between real high-N.A. microscope and low-N.A. microscope images, in addition to averaging, there may be some distortion caused by optical components. Although we did not test our network on real experimental low-N.A. microscope data, we have confidence that the CNN can realize the mapping between LR images and high-resolution images of real data together with learning and correcting the aberration effects of low-N.A. microscopes. Hence, we believe this approach can be used to transfer images that are acquired under LR systems into high-resolution and wide-field images for prescreening applications, significantly extending the space bandwidth product of the output images.
